# Pharmacokinetics and pharmacodynamics of intranasal remimazolam—a randomized controlled clinical trial

**DOI:** 10.1007/s00228-020-02984-z

**Published:** 2020-09-04

**Authors:** Marija Pesic, Frank Schippers, Rob Saunders, Lyn Webster, Martin Donsbach, Thomas Stoehr

**Affiliations:** 1 PAION Deutschland GmbH, Martinstr. 10-12, 52062 Aachen, Germany; 2 Present Address: Creative Clinical Research - CCR GmbH, Wallenroder Straße 7-9, 13435 Berlin, Germany; 3 Early Development Services, Scientific Affairs, PRA Health Sciences, Salt Lake City, UT USA

**Keywords:** Intranasal bioavailability, Pharmacodynamics, Pharmacokinetics, Remimazolam

## Abstract

**Purpose:**

Remimazolam is a novel and ultra-short-acting sedative currently developed for intravenous use in procedural sedation, general anesthesia, and ICU sedation. However, intravenous administration is not always appropriate, depending on the patient or setting. This study evaluated intranasal administration as a potential alternative route.

**Methods:**

The study used a randomized, double-blind, 9 period cross-over design to compare the pharmacokinetics, pharmacodynamics, and safety of single intranasal doses of 10, 20, and 40 mg remimazolam (as powder or solution) with intranasal placebo and 4 mg intravenous remimazolam.

**Results:**

Intranasal remimazolam powder had a consistent absolute bioavailability of approximately 50%; T_max_ was 10 min; AUC and C_max_ were dose-proportional. The higher doses of intranasal solution, however, resulted in decreasing bioavailability and loss of dose-proportionality in AUC and C_max_ despite complete drug absorption due to partial swallowing of dose and the resulting first-pass effect. Pharmacodynamics were generally consistent with PK. Peak effects (drowsiness, relaxation, any, memory, response time) were in similar ranges after intranasal (10 to 40 mg) as intravenous (4 mg) dosing and were partially, but not consistently, dose-related. Safety results were generally consistent with other benzodiazepines; however, intranasal remimazolam (but not placebo) caused nasal discomfort/pain, in some cases even severe.

**Conclusions:**

Intranasal administration of remimazolam was safe and caused sedative effects. However, the severe pain and discomfort caused by intranasal remimazolam prohibit its use by this route of administration, at least with the currently available intravenous formulation.

## Introduction

Remimazolam is a novel, ultra-short-acting benzodiazepine currently being developed for intravenous use for sedation during short medical procedures, the induction and maintenance of general anesthesia, and for ICU sedation [1]. Following intravenous administration, remimazolam is rapidly hydrolyzed to an inactive metabolite, CNS7054, by the activity of carboxylesterase-1, mainly located in the liver. The abundance of metabolizing esterases as well as the lack of pharmacological activity of the main metabolite makes the plasma clearance of the drug high and predictable, which translates to faster recovery of the patient after sedation compared with patients given, e.g., midazolam [2, 3]; safety data are comparable with those of midazolam and also show significant advantages vs propofol (reduced hemodynamic effects, no injection site pain, and the availability of an antagonist) [1].

In some situations and some patient populations, however, intravenous administration may be problematic. Younger pediatric patients, for example, can be anxious and may not cooperate when faced with intravenous injection. Moreover, in an emergency/trauma setting, intravenous access is sometimes not easily available. In those situations, alternative routes of administration, such as oral or intranasal, can be indispensible [4, 5]. Oral bioavailability of remimazolam is very low, making it an unsuitable alternative route of administration for this drug [6]; this trial was therefore designed as a proof-of-concept, to assess the bioavailability and pharmacokinetic/pharmacodynamic profile of intranasal remimazolam and used the IV drug product for both the IV and IN routes of administration.

## Methods

The trial was conducted in accordance with the declaration of Helsinki and the International Conference on Harmonisation (ICH) E6 Guidelines on Good Clinical Practice at PRA-Early Development Services in Salt Lake City, UT, USA. An Institutional Review Board (Schulmann Associates IRB, Inc.) reviewed and approved the trial protocol and related documents, and all subjects gave their informed consent in writing before any trial procedures were performed. The trial was registered at clinicaltrials.gov as NCT03329014.

### Study design

The study was a randomized, double-blind (not double-dummy), placebo and active comparator nine-period cross-over design. The main objective of the study was to assess the feasibility, PK, PD, and safety of intranasal administration. It was planned for 10 healthy male volunteers aged 18–45 years, with BMI of 19.0–33 kg/m^2^ to complete the study. Therefore, 12 subjects were initially enrolled in order to account for possible dropouts. Furthermore, because intranasal administration is not always easy, it was decided to include only subjects with experience of intranasal drug administration within the previous 12 months (but not in the last 2 weeks) prior to randomization. Subjects with a history of benzodiazepine use within 4 weeks prior to randomization, alcohol abuse or drug addiction, who failed a drug/alcohol screening test, or who had abnormal ECG readings were excluded.

Eligible subjects were admitted on day − 1. Computerized randomization was performed on an even distribution basis to 1 of 10 treatment sequences, with each sequence consisting of 9 treatment periods. Each sequence began with remimazolam 4 mg IV administered over 1 min and was followed by single doses of 10, 20, or 40 mg remimazolam or 20 mg placebo, each of these administered as powder and solution such that (1) doses of powder or solution, respectively, were each administered in order of ascending dose (i.e., 10, 20, 40 mg); however, higher doses of powder could precede lower doses of solution and vice versa, and (2) placebo doses were administered in treatment periods 2 to 6. It is also important to note that the IN powder was exactly as formulated in the lyophilized IV drug product, and the IN solution was the same IV powder drug product, but reconstituted in the lowest feasible volume of water for injection. Therefore, higher doses translated to larger amounts of formulated drug in each nostril (41, 82, or 165 mg powder/nostril) or volumes (0.43, 0.85, or 1.7 mL solution/nostril). The IN powder formulation was sniffed by the subjects using a straw, dividing the dose between the left and the right nostril. The IN solution was self-administered by the subjects using an intranasal dosing syringe. The starting 10 mg dose for intranasal application was chosen based on considerations of local and systemic tolerability, as well as a predicted intranasal bioavailability between 10 and 50%. Intranasal studies in rats and minipigs indicated that doses of 50–60 mg are locally safe. Based on the same nonclinical studies, the predicted intranasal bioavailability was ~ 10%; however, literature on the IN bioavailability of other benzodiazepines indicated that bioavailability could be up to 50%. Assuming this as a worst case bioavailability, an intranasal dose of 10 mg would correspond to an IV dose of 5 mg, a dose that has shown good tolerability in phase III trials.

Treatments were administered as single IV or IN doses divided between left and right nostrils, administered in the morning and followed by a 48-h washout period before the next treatment period began. If subjects were deemed fit and free of PD effects 2 h after completion of all assessments on day 17, they were discharged from the clinic. A follow-up was performed 3 ± 2 days after the final treatment. The maximum period of subject participation in the trial was therefore 50 days, and inpatient time was up to 18 days.

### PD measures

As shown in Appendix Table 3, PD parameters included bipolar measures of alertness/drowsiness and agitation/relaxation, as well as unipolar measures of “any effects,” using visual analogue scales (VAS) ranging from 0 to 100. For bipolar measures, 50 was marked as neutral in the scale (e.g., “neither agitated nor relaxed”); for unipolar measures, 0 represented “not at all” and 100 “extremely.” Effects upon visual memory and learning were assessed using paired associates learning (PAL) [7, 8], and effects upon response times were measured using the simple reaction time test from the CANTAB RTI [9].

Pharmacodynamic (PD) assessments were performed at screening and in each treatment period at pre-dose, and at various time points from 5 to 180 min post-dose. Alertness/drowsiness and agitation/relaxation assessments were performed pre-dose and 5, 10, 30, 60, and 180 min post-dose. PAL and RTI tests were performed pre-dose and 10 (only PAL), 20 (only RTI) 30, 60, and 180 min post-dose.

Evaluation of PD effects was based on peak effect (E_max_, except for the bipolar measures of sedation, which used E_min_), time to peak effect, and time-averaged effect (i.e., area under the time effect curve (AUEC)) and included data from all subjects who completed all 9 treatment periods.

### PK

Venous blood samples were collected pre-dose and at 2, 5, 10, 20 (intranasal administration only), 30, 60, 90 (intranasal administration only), 180, and 240 min post-dose for determination of plasma concentrations of remimazolam or CNS7054, the inactive metabolite of remimazolam. PK endpoints, including C_0_ (IV only), C_max_, T_max_, AUC, and *t*_1/2_ were calculated using a non-compartmental model in Phoenix® WinNonlin and the evaluation included data from all subjects who received at least 1 dose of trial medication and had sufficient concentration-time data to permit calculation of at least 1 key parameter (C_max_, AUC_0-last_, or AUC_0-inf_). Plasma concentrations below the limit of quantification (2 ng/mL for remimazolam, 20 ng/mL for CNS7054) were replaced with zero. Descriptive statistics were only reported where ≥ 50% of subjects had values above the lower limit of quantification.

### Safety

Standard safety parameters were assessed including adverse event (AE) questioning and spontaneous reporting (incidence, severity, treatment-relatedness), laboratory parameters (hematology, clinical, urinalysis), vital signs (heart rate, blood pressure, respiration, O_2_ saturation, oral temperature), 12-lead ECG, and physical examination. In addition, suicidal behavior was assessed using the Columbia-Suicide Severity Rating Scale (C-SSRS), the nose and throat were examined by a qualified provider, and subjects completed a Nasal Effects Questionnaire (IN treatment periods only) which included a subjective evaluation of “need to blow nose,” “burning,” “congestion,” “irritation,” “pain,” “runny nose,” and “throat” using a scale ranging from “none” (score of 0) through “severe” (score of 3) as well as a VAS scoring of pain (see Appendix Table 3).

### Statistics

Sample size determination was based on the precedent of previous studies with intranasal administration of drugs [10], and no formal sample size calculation was performed. Therefore, only descriptive data are presented. Analyses of safety included all treated subjects, while analyses of PD effects included those subjects who completed all treatment phases. Since all treated subjects had at least 1 recorded plasma concentration, all subjects were included in analyses of PK.

For the PD analysis, a mixed effects linear model in SAS® was used for analysis of variance. As the PD parameters were determined to be normally distributed using a Shapiro-Wilk *W* test, treatment comparisons were presented as least squared means, difference between means, and 95% confidence intervals. There were no adjustments for multiplicity.

## Results

Of 26 male subjects screened, 12 subjects were randomized, received at least 1 dose of trial medication, and were included in the safety and PK analyses. Two subjects discontinued early: 1 due to an AE (toothache, not considered drug-related), and 1 due to own withdrawal of consent, leaving 10 who completed the trial and were included in analyses of pharmacodynamics (PD).

Almost all randomized subjects (11/12, 92%) were white and 1 (8%) was American Indian or Alaskan native. The mean (± SD) age was 27.2 ± 4.76 and the median BMI was 22.8 (range 20.7 to 27.8). In all, 11 of 12 subjects (92%) confirmed experience with IN administration of dry-powder drugs, and although the remaining subject confirmed IN drug use in the previous 12 months at screening, further details were not collected. Drug and alcohol screening was negative for all subjects at admission (day − 1)), apart from cannabinoid use (positive for 10/12 (83%)) which was permitted at the Investigator’s discretion.

### PK

**Fig. 1:**
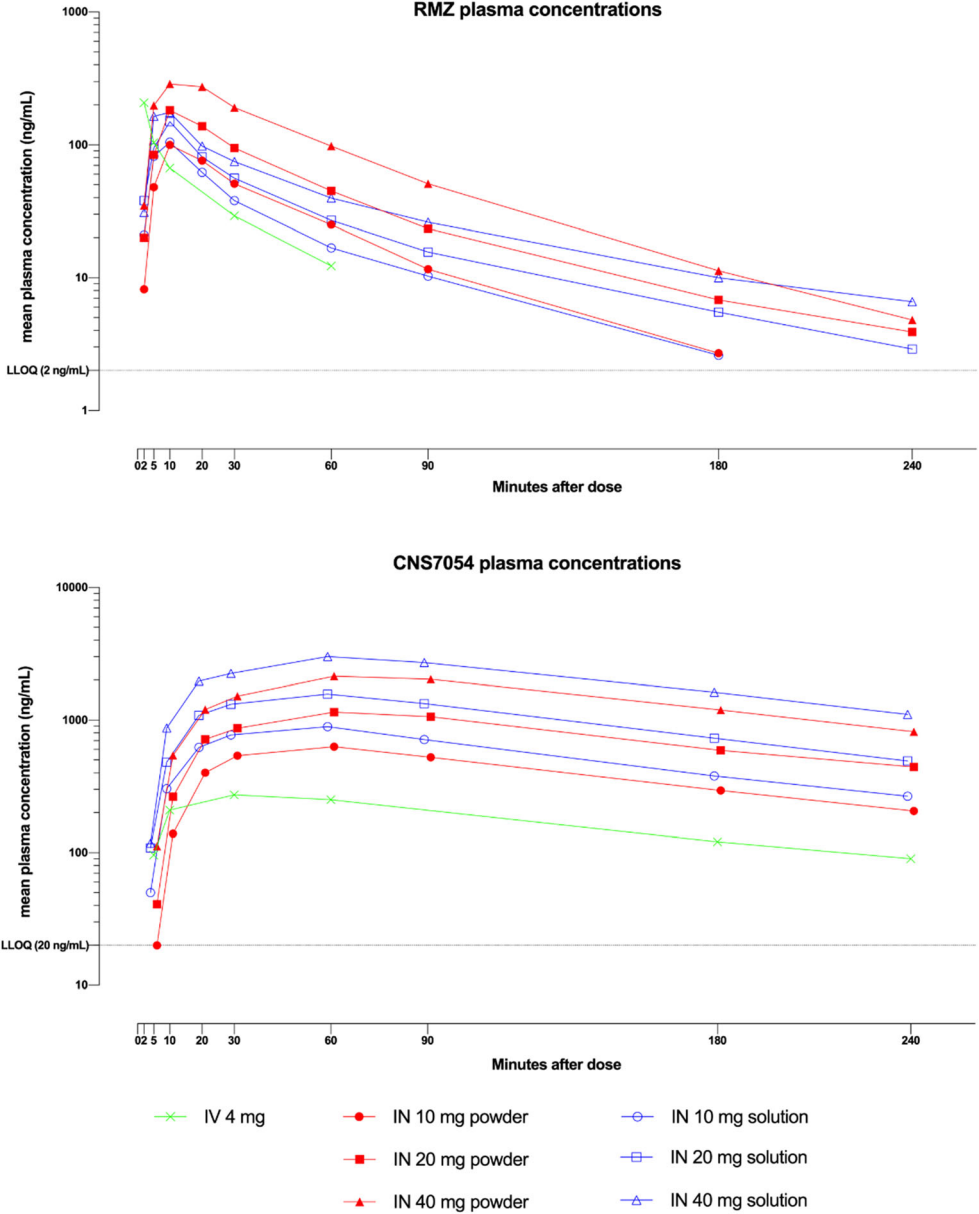
Plasma concentrations of remimazolam and its main metabolite (CNS7054) after intranasal vs intravenous dosing

Remimazolam 4 mg IV resulted in a C_max_ of 213 ng/mL with a terminal elimination half-life of approximately 30 min. After IN dosing with both powder and solution, C_max_ was reached after approximately 10 min, was dose-dependent and ranged from 108 to 330 ng/mL (10 to 40 mg IN powder) and 112 to 209 ng/mL (10 to 40 mg IN solution) (Table 1, Fig. 1).

**Table 1:** Summary of key PK parameters for remimazolam and CNS7054

Parameter	IV	IN powder			IN solution		
Dose	4 mg	10 mg	20 mg	40 mg	10 mg	20 mg	40 mg
*n*	12	11	11	10	11	11	10
Remimazolam (active parent compound)							
C_max_ (ng/mL), mean ± SD	213 ± 144	108 ± 44	188 ± 59	330 ± 90	112 ± 36	165 ± 106	209 ± 100
T_max_ (h), median (min–max)	0.03 (0.017, 0.083)	0.167 (0.167, 0.333)	0.167 (0.083, 0.333)	0.175 (0.083, 0.367)	0.167 (0.083, 0.183)	0.167 (0.083, 0.167)	0.167 (0.083, 0.333)
AUC_(0-last)_ (ng h/mL), mean ± SD	55 ± 16	69 ± 20	135 ± 42	262 ± 80	60 ± 25	93 ± 37	133 ± 15
AUC_(0-inf)_ (ng h/mL), mean ± SD	59 ± 16	72 ± 19	141 ± 45	268 ± 83	62 ± 26	98 ± 39	144 ± 16
*t*_1/2_, (h), mean ± SD	0.49 ± 0.19	0.66 ± 0.20	0.87 ± 0.19	0.69 ± 0.11	0.71 ± 0.27	0.97 ± 0.19	1.17 ± 0.18
F (%), mean ± SD	NA	49 ± 12	49 ± 18	48 ± 22	47 ± 23	34 ± 9.8	26 ± 8.7
Statistical comparison of bioavailability expressed as relative exposure to parent drug for IN vs IV							
Relative exposure vs IV (%) (C_max_/D), LS mean (90% CI)	NA	0.22 (0.14, 0.30)	0.20 (0.15, 0.27)	0.18 (0.23, 0.24)	0.24 (0.18, 0.32)	0.16 (0.12, 0.21)	0.11 (0.08, 0.14)
Relative exposure vs IV (%) AUC_(0-last)_/D, LS mean (90% CI)	NA	0.49 (0.42, 0.58)	0.48 (0.41, 0.57)	0.47 (0.40, 0.56)	0.42 (0.36, 0.50)	0.34 (0.29, 0.40)	0.25 (0.21, 0.29)
CNS7054 (inactive metabolite)							
C_max_ (ng/mL), mean ± SD	275 ± 41	655 ± 103	1191 ± 217	2300 ± 556	960 ± 172	1710 ± 361	3572 ± 1221
T_max_ (h), median (min–max)	0.5 (0.167–1.00)	1.0 (0.500–1.50)	1.0 (1.00–1.50)	1.0 (1.00–1.50)	1.0 (0.500–1.02)	1.0 (0.500–1.50)	1.0 (0.333–3.00)
AUC_(0-last)_ (ng h/mL), mean ± SD	703 ± 121	1553 ± 236	3003 ± 598	5678 ± 1352	2143 ± 293	3913 ± 701	7871 ± 1741
AUC_(0-inf)_ (ng h/mL), mean ± SD	NC ^a)^	1571 ± NC	2729 ± NC	6560 ± NC	2626 ± 205	2915 ± 548	9422 ± 370
*t*_1/2_ (h), mean ± SD	NC ^a)^	1.32 ± NC	1.37 ± NC	1.40 ± NC	1.52 ± 0.14	1.30 ± 0.11	1.29 ± 0.04
Statistical comparison of bioavailability expressed as relative exposure to metabolite for IN vs IV							
Relative exposure vs IV (%) (C_max_/D), LS mean (90% CI)	NA	0.94 (0.84, 1.04)	0.85 (0.76, 0.95)	0.81 (0.72, 0.91)	1.40 (1.25, 1.56)	1.24 (1.11, 1.38)	1.23 (1.09, 1.37)
Relative exposure vs IV (%) AUC_(0-last)_/D, LS mean (90% CI)	NA	0.87 (0.80, 0.94)	0.83 (0.77, 0.90)	0.78 (0.73, 0.85)	1.23 (1.14, 1.33)	1.12 (1.03, 1.20)	1.09 (1.01, 1.18)

*AUC* area under the plasma concentration time curve, *C*_*max*_ maximum observed plasma concentration, *F* bioavailability, *IN* intranasal, *IV* intravenous, *RMZ* remimazolam, *T*_*max*_ time to C_max_, *t*_*1/2*_ elimination half-life

^a^For subjects with %AUC_ext_ > 20%, the following parameters are not summarized: AUC_0-inf_, AUC_0-inf_/D, and *t*_1/2_

After dosing with IN powder, exposure (AUC and C_max_) to remimazolam was dose proportional and absolute bioavailability (F%) was ca. 50% (49%, 49% and 48% for 10, 20, and 40 mg, respectively) while relative C_max_ vs IV (C_max_/dose), was ca. 20% (22%, 20%, and 18% for 10, 20, and 40 mg, respectively) at all doses. The elimination half-life was independent of dose (0.7 to 0.9 h). For the metabolite CNS7054, C_max_ was generally reached after ca. 1 h and exposure (C_max_ and AUC) was dose proportional. Relative exposure to the metabolite after IN powder as compared to IV administration was high (81 to 94% based on C_max_/dose; 78 to 87% based on AUC/dose). This indicates nearly complete absorption, defined as relative exposure of 100%, which decreased minimally with increasing dose. The terminal elimination half-life was ca. 1.3–1.4 h for all doses.

After dosing with IN solution, exposure was less than dose proportional for remimazolam and absolute bioavailability decreased from 47 to 26% (F%) and relative C_max_ concentrations from 24 to 11% (C_max_/dose) with increasing dose. The elimination half-life increased with dose (and therefore dose volume) from 0.7 to 1.2 h. For the metabolite, however, C_max_ was generally reached within ca. 1 h, and exposure was dose-proportional. Relative exposure to the metabolite after IN solution as compared to IV administration was high, indicating complete absorption but exposure also decreased slightly with increasing dose from 140 to 123% (C_max_/dose) and 123 to 109% (AUC/dose).

### Pharmacodynamics

#### Sedation (drowsiness and relaxation, bipolar VAS)

**Fig. 2:**
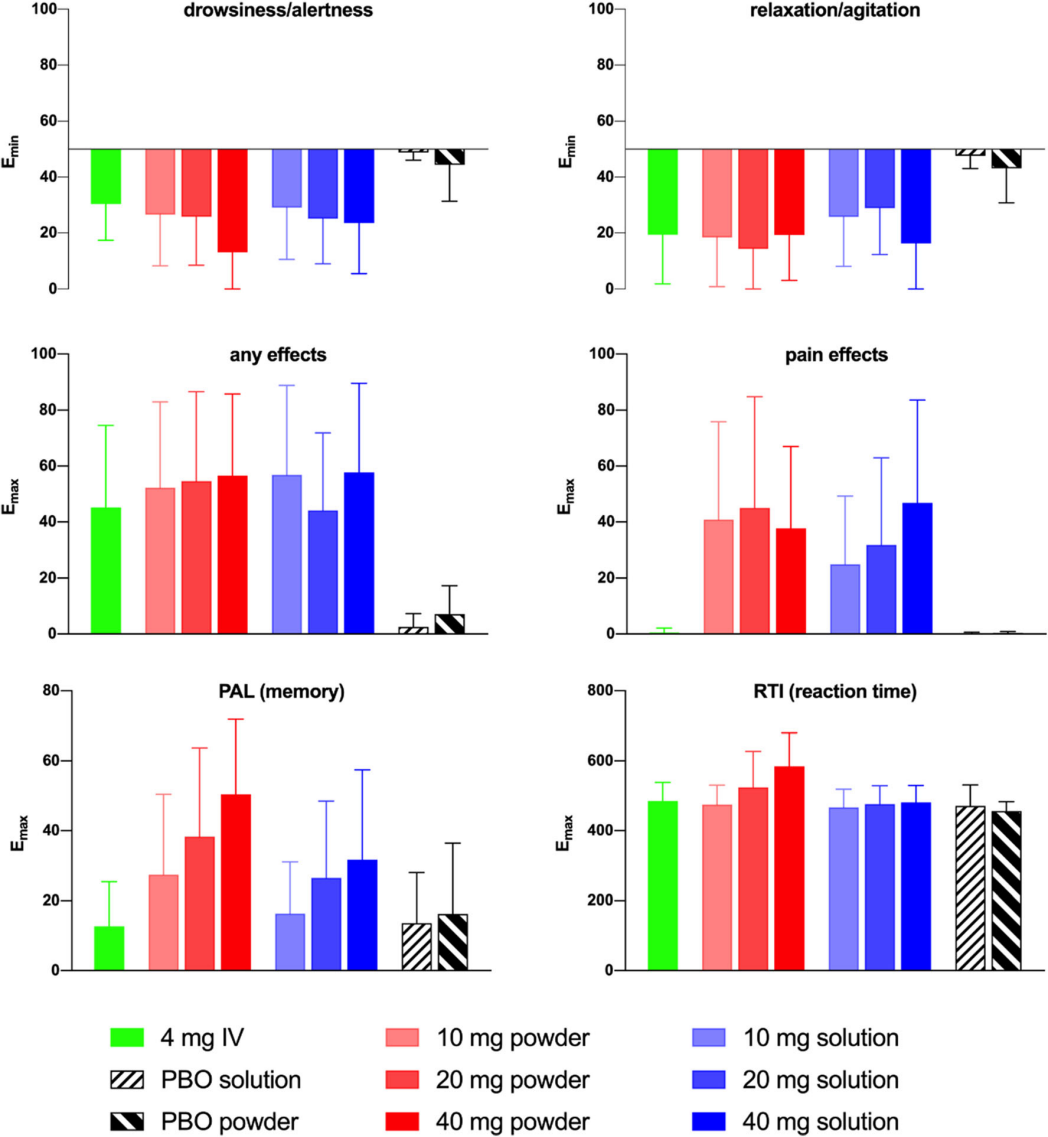
Comparison of peak remimazolam PD effects (IV vs IN vs placebo)

As shown in Fig. 2, compared to IN placebo, remimazolam consistently induced significant drowsiness and relaxation by both the IV and IN routes at all doses (Table 2), despite large variabilities in absolute values (Appendix Table 4). The peak effects (E_min_) were achieved within 10 to 20 min for lower doses and after over 30 min for the 40 mg doses and these were in the similar range for the IN and IV administration routes at all tested doses. A weak dose-response was apparent in drowsiness results for both powder and solution, and the 40 mg dose led to markedly stronger drowsiness for IN powder and markedly stronger relaxation for IN solution, respectively. Generally, relaxation and drowsiness effects appeared to be slightly weaker for the IN solution vs the IN powder. The time-average (AUEC) sedation effects from IN dosing were considerably stronger for the 20 and 40 mg doses vs 10 mg and were slightly stronger than the 4 mg IV.

**Table 2:** Summary of relative peak, time to peak, and time-averaged PD effects vs placebo (completer set)

Treatment, dose	VAS measures				Memory/reaction time measures	
	Alertness/drowsiness	Agitation/relaxation	Any effects	Pain effects	PAL	RTI
**Parameter**	**E**_**min**_	**E**_**min**_	**E**_**max**_	**E**_**max**_	**E**_**max**_	**E**_**max**_
	**Δ Peak effect (E**_**max**_ **or E**_**min**_**) vs placebo: LS Mean (95% CI)**					
RMZ 4 mg IV						
vs PBO powder IN	− 14.0 (− 23.9, − 4.1)	− 23.7 (− 33.9, − 13.5)	38.1 (23.6, 52.6)	0.1 (− 18.6, 18.8)	− 3.5 (− 15.2, 8.2)	29.2 (− 10.8, 69.2)
vs PBO solution IN	− 18.4 (− 28.3, − 8.5)	− 28.2 (− 38.4, − 18.0)	42.7 (28.2, 57.2)	0.3 (− 18.4, 19.0)	− 0.9 (− 12.6, 10.8)	14.6 (− 25.4, 54.6)
RMZ powder IN						
10 mg vs PBO powder	− 17.9 (− 27.8, − 8.0)	− 24.7 (− 34.9, − 14.5)	45.2 (30.7, 59.7)	40.5 (21.8, 59.2)	11.2 (− 0.5, 22.9)	18.5 (− 21.5, 58.6)
20 mg vs PBO powder	− 18.6 (− 28.5, − 8.7)	− 28.7 (− 38.9, − 18.5)	47.5 (33.0, 62.0)	44.6 (25.9, 63.3)	22.1 (10.4, 33.8)	68.3 (28.3, 108.3)
40 mg vs PBO powder	− 31.2 (− 41.1, − 21.3)	− 23.8 (− 34.0, − 13.6)	49.5 (35.0, 64.0)	37.3 (18.6, 56.0)	34.2 (22.5, 45.9)	127.9 (87.9, 167.9)
RMZ solution IN						
10 mg vs PBO solution	− 19.8 (− 29.7, − 9.9)	− 21.9 (− 32.1, − 11.7)	43.4 (28.9, 57.9)	24.7 (6.0, 43.4)	2.8 (− 8.9, 14.5)	− 3.7 (− 43.7, 36.3)
20 mg vs PBO solution	− 23.7 (− 33.6, − 13.8)	− 18.7 (− 28.9, − 8.5)	41.6 (27.1, 56.1)	31.5 (12.8, 50.2)	12.9 (1.2, 24.6)	5.3 (− 34.7, 45.3)
40 mg vs PBO solution	− 25.3 (− 35.2, − 15.4)	− 31.3 (− 41.5, − 21.1)	55.2 (40.7, 69.7)	46.7 (28.0, 65.4)	18.1 (6.4, 29.8)	10.4 (− 29.7, 50.4)
**Parameter**	**AUEC**_**(0–180)**_	**AUEC**_**(0–180)**_	**AUEC**_**(0–180)**_	**AUEC**_**(0–180)**_	**AUEC**_**(0–180)**_	**AUEC**_**(0–180)**_
	**Δ Time-averaged effect (AUEC**_**0–180**_**) vs placebo, LS Mean (95% CI)**					
RMZ 4 mg IV						
vs PBO powder IN	390 (− 962, 1741)	− 154 (− 1459, 1152)	1604 (528, 2681)	− 10.5 (− 1036, 1015)	− 2228 (− 2553, − 903)	335 (− 4338, 5007)
vs PBO solution IN	− 678 (− 2029, 674)	− 979 (− 2285, 326)	1804 (728, 2881)	5.7 (− 1020, 1031)	− 1732 (− 3057, − 407)	1248 (− 3425, 5920)
RMZ powder IN						
10 mg vs PBO powder	787 (− 565, 2138)	− 25.6 (− 1331, 1280)	1962 (886, 3039)	1172 (146, 2198)	− 1052 (− 2377, 273)	19.5 (− 4653, 4692)
20 mg vs PBO powder	− 513 (− 1905, 879)	− 1286 (− 2630, 58.4)	3171 (2062, 4280)	1554 (497, 2610)	540 (− 824, 1904)	5725 (1052, 10,397)
40 mg vs PBO powder	− 1553 (− 2905, − 202)	− 1144 (− 2450, 162)	3530 (2454, 4606)	2087 (1061, 3113)	125 (− 1200, 1450)	6884 (2212, 11,557)
RMZ solution IN						
10 mg vs PBO solution	− 140.7 (1491, 1211)	− 976 (− 2281, 330)	1763 (686, 2839)	832 (− 194, 1857)	− 676 (− 2001, 649)	3083 (− 1589, 7756)
20 mg vs PBO solution	− 1494 (− 2846, − 143)	− 2319 (− 3624, − 1013)	2284 (1208, 3360)	795 (− 231, 1820)	41.3 (1284, 1366)	6190 (1518, 10,863)
40 mg vs PBO solution	− 1182 (− 2533, 169)	− 1253 (− 2559, 52.4)	2397 (1321, 3473)	945 (− 81.1, 1970)	621 (− 704, 1946)	1670 (3003, 6342)

*AUEC* area under the effect time curve, *IN* intranasal, *IV* intravenous, *PAL* paired associates learning, *PBO* placebo, *RMZ* remimazolam, *RTI* reaction time index

#### Any effects (unipolar VAS)

IN dosing with remimazolam at 10 to 40 mg dose led to significantly greater peak scores for “any effect” than IN placebo, as shown in Fig. 2 and Table 2. Peak scores were generally reached in 10 to 20 min, T_max_ appeared dose-dependent after IN powder dosing, but not after IN solution. Peak effects were strongest for the IN powder (52.3 to 56.6) vs solution (44.1 to 57.7) and weakest for IV dosing (45.2). Peak scores for IN administration did not appear to increase notably with dose. Time-averaged effects were stronger overall for powder vs comparable doses of the solution and also stronger after both IN 20 mg and 40 mg doses vs 10 mg.

#### Memory

Mean peak PAL total error scores were unaffected by remimazolam 4 mg IV vs placebo (powder and solution), indicating no effect upon visual memory. IN dosing of remimazolam led to dose-dependent visual memory impairment vs placebo in mean peak scores at all tested doses, particularly at the 20 and 40 mg doses, which were more pronounced with powder, reaching peak effect (E_max_) after a median of 10 to 20 min (Fig. 2). Time-averaged effects upon total error scores were weak and highly variable and were dose dependent for the solution but not the powder (Table 2).

#### Reaction time

RTI scores were not affected by remimazolam 4 mg IV vs placebo. IN powder led to weak, dose-dependent increases in peak RTI scores at the 20 and 40 mg doses (Fig. 2), which were achieved within a median of 30 to 40 min post-dose and resolved within an hour. No significant effect was observed upon peak scores following administration of IN solution. At the higher doses (20 and 40 mg), time-average effects of IN powder were markedly increased vs remimazolam 4 mg IV.

### Safety

Overall, adverse events (AEs) were reported by all subjects in at least 1 treatment period during the trial and were more common after active treatment (58 to 100%) than after placebo (27%). AEs were more common after IN (64 to 100% of subjects) dosing than after IV remimazolam 4 mg (58%) and appeared to be dose related (see Appendix Table 5). The incidences of AEs following active treatment were consistent with the use of benzodiazepines and were most common in the system organ classes Investigations (increases/decreases in blood pressure: remimazolam, up to 46% vs placebo, up to 9%), nervous system disorders (e.g., somnolence: remimazolam, up to 40% vs placebo, 0%), and psychiatric disorders (e.g., euphoric mood: remimazolam, up to 40% vs placebo, up to 9%). No subjects had serious AEs and only 1 subject discontinued after treatment with placebo (due to moderate toothache). All AEs were mild or moderate except for 1 subject with severe nasal discomfort after treatment with remimazolam 10 mg solution.

Transient increases in heart rate and mean arterial pressure were seen approximately 2 to 5 min after active treatment. No abnormal trends were apparent in clinical laboratory parameters, ECG, respiratory rate, O_2_ saturation, vital signs or physical examination and no subjects experienced suicidal ideation.

### Pain and nasal effects

Overall, in response to the Nasal Effect Questionnaire, mild to moderate mean discomfort was reported for all categories. Within 5 min after IN administration of remimazolam (powder or solution), up to 1–2 subjects at each dose reported severe pain, burning, irritation, congestion, blow, runniness, and throat effects, respectively (Appendix Table 5) at all doses except 40 mg solution. Discomfort was not reported following administration of either powder or solution placebo. Overall, discomfort was not dose proportional and resolved within 30 to 180 min. These results are in line with the observed VAS pain scores in which neither the IN placebo treatments nor the remimazolam 4 mg IV resulted in notable mean pain scores after administration (placebo powder 0.4, placebo solution 0.2, remimazolam IV 0.5). In contrast, IN administration of remimazolam led to significant increases in mean peak pain scores. Pain effects reported after IN dosing of remimazolam were comparable between powder (range 37.7 to 45.0) and solution (range 24.9 to 46.9). The effects seem to be dose-related for solution, but not for the powder (Fig. 2). Peak pain scores reached maximum levels with 6 to 15 min for most subjects and resolved within an hour (except for 40 mg powder group). Time-averaged pain scores appeared to be dose-dependent for IN powder and were also elevated for the 40 mg IN solution. No clinically significant abnormalities were reported for nose and throat examinations.

## Discussion

This trial was designed to evaluate the clinical potential for intranasal administration of remimazolam. As a proof-of-concept, the intravenous formulation was used, with no optimization. This formulation is a lyophilized powder containing ~ 17% remimazolam besylate, 33% lactose, and 50% dextran 40. Single doses of 10 to 40 mg remimazolam were given intranasally as either lyophilized powder or as reconstituted solution, and the effects were compared with those of a single 4 mg intravenous dose or nasal placebo (powder and solution).

Intranasal remimazolam, either as powder or solution, resulted in rapid, dose-related increases in the plasma concentrations, reaching levels sufficient to induce sedative effects of drowsiness and relaxation. C_max_ was reached in approximately 10 min for both powder and solution, and absolute bioavailability (AUC/dose) was 48 to 49% for the powder and independent of dose. The increasing dose volumes of the solution (from 0.43 up to 1.7 mL in each nostril for the 10 to 40 mg doses, respectively) resulted in reduced intranasal bioavailability at higher solution doses (from 47% for 10 mg to 26% for 40 mg). The most likely reason for dose dependent reduction of BA is the swallowing of excess fluid which was more prominent at higher doses/volumes. Despite almost complete uptake, oral bioavailability of remimazolam is low due to rapid first pass metabolism. This also explains the increased concentrations of metabolite following IN dosing of solution.

The PD effects of intranasal remimazolam were consistent with the observed PK; they were apparent within 5 min of administration and reached a peak within 10 to 20 min. Consistent with the observed PK, the magnitude and dose relationship of PD responses were weaker for solution than for the powder. Moreover, PD effects seemed to reach the maximum a bit later and to last longer following IN administration of a powder than a solution especially at higher doses as reflected by an increase in T_max_ and AUEC. One could speculate that large amounts of dextran and lactose in the IV formulation enhanced retention of powder on the nasal mucosa extending the period of uptake instead of the drug being swallowed, as was apparently the case for the solution.

In pediatric patients, the distress associated with the establishment of intravenous sedation, i.e., intravenous access can represent a considerable obstacle to optimal treatment. Assuming suitable absorption kinetics, intranasal administration can be a highly desirable alternative to the intravenous route, circumventing lack of cooperation. In this trial, intranasal remimazolam demonstrated onset within 5 min of administration and achieved peak drowsiness and relaxation within 10 to 20 min. The reported drowsiness and relaxation responses in this trial demonstrate that sedation via intranasal dosing with remimazolam is feasible, even with a non-optimized formulation. Onset times were faster than those typically reported for midazolam, which is used intranasally with some success, e.g., for imaging or in dental practices [11–15]. A limitation of the current trial design was the use of non-optimized IV formulation, resulting in the need to apply large volumes of either solution or powder in order to achieve clinical effects. These volumes were in both cases beyond what has been reported as optimal for nasal delivery [16, 17], which could have resulted in lower absorption and bioavailability even at low doses. Therefore, while this trial clearly shows a low potential of the current formulation to be abused via the intranasal route, it offers no more than a proof of concept on the potential clinical use of the intranasal route for therapeutic purposes.

Remimazolam, administered IV, as IN powder, or as IN solution was safe and generally well tolerated looking at systemic effects. However, local tolerability of intranasal remimazolam showed significant, even severe discomfort in a small number of subjects though without relevant findings in the visual nose and throat examination. Although not apparently dose-related, the lack of local intolerance to the pH-matched placebo suggests that this pain was not caused by pH of the formulation but was more likely caused by remimazolam itself.

The clinical need for intranasal sedation is given by fear of injection, particularly in younger patients. Therefore, to be a clinically useful alternative, intranasal treatment must be demonstrably at least as tolerable as intravenous injection [14]; something which the current intravenous formulation does not seem to offer when administered intranasally: it causes considerable local discomfort (thus negating the purpose of the intranasal route).

Beyond the intended indication, benzodiazepines are known to have significant human abuse potential [18] and the potential for the human abuse of intravenous remimazolam has already been addressed in a separate trial (CNS7056-014, in publication). The results showed a low overall intravenous abuse potential, broadly comparable with that of the short-acting benzodiazepine comparator midazolam (Schippers et al., submitted). However, in the proof-of-concept trial reported here, although confirmation of PD responses following intranasal administration may appear to support a potential for abuse, the degree of discomfort/pain reported by subjects as well as very large amounts of powder needed for achieving PD effects, suggests that abuse of remimazolam by this route is unlikely, especially considering the short duration of positive effects, in this case shown by drowsiness and relaxation.

In conclusion, this proof-of-concept trial showed that intranasal administration of 10 to 40 mg remimazolam as powder or solution is safe and partially efficacious, reaching levels sufficient to induce rapid sedation. However, difficulties with intranasal dosing volume (solution) and the degree of local discomfort observed shortly after administration suggest that remimazolam is unsuitable for this mode of treatment, at least in its current formulation for intravenous application.

## Appendix

**Table 3:** Description of PD parameters

Parameter	Scale	Question	Minimum	Neutral	Maximum
Safety, local tolerance measures					
Pain	VAS, unipolar	At this moment, I feel pain or discomfort in my nose:	0 (not at all)	-	100 (extremely)
Pharmacodynamic effects					
Any effects	VAS, unipolar	At this moment, I feel any drug effects:	0 (not at all)	-	100 (extremely)
Alertness/drowsiness	VAS, bipolar	At this moment, my mental state is:	0 (very drowsy)	50	100 (very alert)
Agitation/relaxation	VAS, bipolar	At this moment, my mood is:	0 (very relaxed)	50	100 (very agitated)
PAL	Total error score	NA	0	NA	NA

*NA* not applicable

**Table 4:** Summary of absolute peak, time to peak, and time-averaged PD effects (completer set)

Treatment, dose	VAS measures				Memory/reaction time measures	
	Alertness/drowsiness	Agitation/relaxation	Any effects	Pain effects	PAL	RTI
**Parameter**	**E**_**min**_	**E**_**min**_	**E**_**max**_	**E**_**max**_	**E**_**max**_	**E**_**max**_
	**Peak effect (E**_**max**_ **or E**_**min**_**): mean ± SD**					
PBO						
Powder	44.4 ± 13.0	43.1 ± 12.3	7.1 ± 10.2	0.4 ± 0.5	16.2 ± 20.2	456 ± 27.0
Solution	48.8 ± 2.8	47.6 ± 4.6	2.5 ± 4.8	0.2 ± 0.4	13.6 ± 14.4	471 ± 60.1
RMZ IV						
4 mg	30.4 ± 14.8	19.4 ± 17.5	45.2 ± 29.3	0.5 ± 1.6	12.7 ± 12.7	485 ± 53.2
RMZ IN powder						
10 mg	26.5 ± 18.1	18.4 ± 17.5	52.3 ± 30.6	40.9 ± 35.0	27.4 ± 23.0	474.8 ± 55.4
20 mg	25.8 ± 17.3	14.4 ± 14.6	54.6 ± 31.9	45.0 ± 39.7	38.3 ± 25.4	524.6 ± 102.2
40 mg	13.2 ± 13.7	19.3 ± 16.2	56.6 ± 29.1	37.7 ± 29.3	50.4 ± 21.5	584.2 ± 95.7
RMZ IN solution						
10 mg	29.0 ± 18.4	25.7 ± 17.6	45.9 ± 31.9	24.9 ± 24.4	16.4 ± 14.7	467.2 ± 52.1
20 mg	25.1 ± 16.1	28.9 ± 16.5	44.1 ± 27.8	31.7 ± 31.2	26.5 ± 22.0	476.2 ± 52.6
40 mg	23.5 ± 18.0	16.3 ± 16.7	57.7 ± 31.8	46.9 ± 36.6	31.7 ± 25.7	481.2 ± 48.7
**Parameter**	**T**_**min**_	**T**_**min**_	**T**_**max**_	**T**_**max**_	**T**_**max**_	**T**_**max**_
	**Time to peak effect (T**_**max**_ **or T**_**min**_**, min): median [Q1, Q3]**					
PBO						
Powder	19.7 [0.0, 32.3]	9.9 [0.0, 32.4]	9.8 [8.1, 12.3]	0.0 [0.0, 6.8]	24.7 [12.6, 185]	16.5 [0.0, 92.6]
Solution	0.0 [0.0, 17.9]	3.3 [0.0, 11.7]	14.2 [8.7, 32.0]	0.0 [0.0, 0.0]	52.1 [37.2, 62.1]	47.6 [0.0, 123]
RMZ IV						
4 mg	10.2 [7.5, 30.1]	12.2 [7.6, 32.5]	7.5 [7.0, 12.1]	0.0 [0.0, 0.0]	0.0 [0.0, 13.0]	106.9 [0.0, 182]
RMZ IN powder						
10 mg	9.3 [7.7, 32.4]	12.1 [8.1, 31.1]	9.9 [8.1, 12.3]	8.1 [7.7, 12.3]	13.4 [12.9, 16.0]	29.1 [0.0, 32.8]
20 mg	18.7 [8.7, 35.7]	14.2 [8.5, 32.0]	14.2 [8.7, 32.0]	8.9 [7.6, 12.4]	13.6 [13.2, 26.6]	32.1 [22.4, 33.5]
40 mg	19.1 [12.2, 32.3]	34.4 [32.2, 62.2]	20.0 [14.3, 36.8]	15.2 [12.5, 36.8]	19.0 [16.8, 25.6]	38.0 [31.2, 62.5]
RMZ IN solution						
10 mg	11.3 [7.6, 12.5]	11.3 [7.6, 12.5]	10.2 [6.9, 12.2]	6.8 [6.1, 9.0]	12.8 [12.1, 36.1]	28.9 [22.3, 62.5]
20 mg	12.2 [7.2, 32.2]	11.6 [6.8, 32.2]	12.2 [7.2, 32.2]	6.7 [6.3, 7.2]	13.1 [12.9, 35.6]	28.3 [22.4, 63.2]
40 mg	11.1 [6.1, 32.3]	11.0 [6.7, 12.3]	12.3 [10.0, 13.5]	6.9 [6.2, 8.2]	14.1 [13.4, 14.7]	22.5 [0.0, 150]
	**Time-averaged effect (AUEC0–180), mean ± SD**					
PBO						
Powder	− 1533 ± 3196	− 945 ± 1661	257 ± 332	17.0 ± 33.4	1162 ± 1670	− 3352 ± 6860
Solution	− 465 ± 1266	− 119 ± 227	57.1 ± 146	0.8 ± 1.7	666 ± 958	− 4265 ± 8372
RMZ IV						
4 mg	− 1143 ± 1114	− 1098 ± 1313	1861 ± 1531	6.4 ± 20.3	− 1066 ± 1359	− 3017 ± 4007
RMZ IN powder						
10 mg	− 746 ± 1850	− 970 ± 2245	2219 ± 1604	1189 ± 1362	110 ± 794	− 3332 ± 4552
20 mg	− 1837 ± 1643	− 2238 ± 1761	3451 ± 2459	1588 ± 1738	1724 ± 3064	2373 ± 4124
40 mg	− 3086 ± 2122	− 2089 ± 1040	3787 ± 2446	2104 ± 2837	1287 ± 1003	3533 ± 2660
RMZ IN solution						
10 mg	− 605 ± 843	− 1095 ± 1217	1820 ± 1681	832 ± 1338	− 9.5 ± 1073	− 1182 ± 3805
20 mg	− 1960 ± 1933	− 2438 ± 2655	2341 ± 2077	796 ± 865	708 ± 1058	1926 ± 6509
40 mg	− 1648 ± 2043	− 1373 ± 898	2454 ± 1955	945 ± 1299	1287 ± 2444	− 2595 ± 7076

*AUEC* area under the effect time curve, *IN* intranasal, *IV* intravenous, *PAL* paired associates learning, *PBO* placebo, *RMZ* remimazolam, *RTI* reaction time index

**Table 5:** Subjects with treatment-emergent adverse events or severe nasal symptoms

System organ class preferred term	IV	IN powder			IN solution			PBO		Overall
	4 mg	10 mg	20 mg	40 mg	10 mg	20 mg	40 mg	Solution	Powder	
	(*N* = 12)	(*N* = 11)	(*N* = 11)	(*N* = 10)	(*N* = 11)	(*N* = 11)	(*N* = 10)	(*N* = 11)	(*N* = 11)	(*N* = 12)
	*n* (%)									
Total subjects with at least 1 TEAE	7 (58.3)	7 (63.6)	9 (81.8)	10 (100)	9 (81.8)	8 (72.7)	10 (100)	3 (27.3)	3 (27.3)	12 (100)
Ear and labyrinth disorders	1 (8.3)	0	0	0	0	0	0	0	0	1 (8.3)
Hyperacusis	1 (8.3)	0	0	0	0	0	0	0	0	1 (8.3)
Eye disorders	0	0	0	1 (10.0)	0	1 (9.1)	1 (10.0)	0	0	2 (16.7)
Eye irritation	0	0	0	0	0	1 (9.1)	0	0	0	1 (8.3)
Diplopia	0	0	0	1 (10.0)	0	0	1 (10.0)	0	0	1 (8.3)
Gastrointestinal disorders	0	0	0	0	0	0	0	1 (9.1)	1 (9.1)	1 (8.3)
Toothache	0	0	0	0	0	0	0	1 (9.1)	0	1 (8.3)
Paraesthesia oral	0	0	0	0	0	0	0	0	1 (9.1)	1 (8.3)
General disorders and administration site conditions	2 (16.7)	0	0	1 (10.0)	1 (9.1)	0	3 (30.0)	1 (9.1)	0	4 (33.3)
Feeling abnormal	2 (16.7)	0	0	0	1 (9.1)	0	2 (20.0)	1 (9.1)	0	3 (25.0)
Feeling of relaxation	0	0	0	1 (10.0)	0	0	1 (10.0)	0	0	1 (8.3)
Infections and infestations	0	0	1 (9.1)	0	0	0	0	0		1 (8.3)
Folliculitis	0	0	1 (9.1)	0	0	0	0	0	0	1 (8.3)
Injury, poisoning and procedural complications	0	0	0	0	0	0	1 (10.0)	0	0	1 (8.3)
Limb injury	0	0	0	0	0	0	1 (10.0)	0	0	1 (8.3)
Investigations	1 (8.3)	2 (18.2)	1 (9.1)	2 (20.0)	4 (36.4)	5 (45.5)	2 (20.0)	1 (9.1)	0	12 (100)
Blood pressure diastolic increased	1 (8.3)	1 (9.1)	1 (9.1)	0	1 (9.1)	3 (27.3)	1 (10.0)	0	0	8 (66.7)
Blood pressure diastolic decreased	0	1 (9.1)	0	1 (10.0)	3 (27.3)	2 (18.2)	0	1 (9.1)	0	8 (66.7)
Blood pressure systolic increased	0	0	0	0	1 (9.1)	0	2 (20.0)	0	0	3 (25.0)
Blood pressure systolic decreased	0	0	0	1 (10.0)	0	0	0	0	0	1 (8.3)
Nervous system disorders	2 (16.7)	0	1 (9.1)	6 (60.0)	1 (9.1)	2 (18.2)	2 (20.0)	1 (9.1)	2 (18.2)	11 (91.7)
Somnolence	0	0	0	5 (50.0)	0	1 (9.1)	2 (20.0)	0	0	8 (66.7)
Headache	0	0	0	0	0	0	1 (10.0)	1 (9.1)	2 (18.2)	3 (25.0)
Sedation	0	0	0	1 (10.0)	0	0	0	0	0	1 (8.3)
Dysgeusia	0	0	0	0	0	1 (9.1)	0	0	0	1 (8.3)
Dizziness	1 (8.3)	0	0	0	0	0	0	0	0	1 (8.3)
Disturbance in attention	1 (8.3)	0	0	0	0	0	0	0	0	1 (8.3)
Cognitive disorder	0	0	1 (9.1)	0	1 (9.1)	0	0	0	0	1 (8.3)
Psychiatric disorders	4 (33.3)	5 (45.5)	6 (54.5)	3 (30.0)	1 (9.1)	4 (36.4)	5 (50.0)	0	1 (9.1)	9 (75.0)
Euphoric mood	2 (16.7)	3 (27.3)	4 (36.4)	2 (20.0)	1 (9.1)	2 (18.2)	4 (40.0)	0	1 (9.1)	7 (58.3)
Illusion	2 (16.7)	1 (9.1)	1 (9.1)	1 (10.0)	0	1 (9.1)	0	0	0	3 (25.0)
Thinking abnormal	0	0	0	0	0	1 (9.1)	0	0	0	1 (8.3)
Hypervigilance	0	0	0	0	0	0	1 (10.0)	0	0	1 (8.3)
Disorientation	0	1 (9.1)	0	0	0	0	0	0	0	1 (8.3)
Communication disorder	0	0	1 (9.1)	0	0	0	0	0	0	1 (8.3)
Respiratory, thoracic and mediastinal disorders		1 (9.1)	2 (18.2)	2 (20.0)	5 (45.5)	2 (18.2)	5 (50.0)	0	0	7 (58.3)
Nasal discomfort	0	1 (9.1)	1 (9.1)	1 (10.0)	5 (45.5)	2 (18.2)	4 (40.0)	0	0	7 (58.3)
Throat irritation	0	0	0	1 (10.0)	2 (18.2)	1 (9.1)	3 (30.0)	0	0	6 (50.0)
Sneezing	0	0	0	1 (10.0)	0	0	1 (10.0)	0	0	1 (8.3)
Paranasal sinus discomfort	0	0	1 (9.1)	0	0	0	0	0	0	1 (8.3)
Nasal congestion	0	0	1 (9.1)	0	0	0	0	0	0	1 (8.3)
Number of subjects by symptom with severe grading on Nasal Effects Questionnaire at 5 min post-dose										
Blow	0	0	0	1	0	0	0	0	0	0
Burning	0	1	2	0	2	1	0	0	0	0
Congestion	0	0	2	0	0	0	0	0	0	0
Irritation	0	1	1	1	1	1	0	0	0	0
Pain	0	0	1	0	0	0	0	0	0	0
Runny	0	0	1	0	0	0	0	0	0	0
Throat	0	1	0	0	1	0	0	0	0	0
